# Serum Insulin-Like Growth Factor-1 in Parkinson's Disease; Study of Cerebrospinal Fluid Biomarkers and White Matter Microstructure

**DOI:** 10.3389/fendo.2018.00608

**Published:** 2018-11-02

**Authors:** Farzaneh Ghazi Sherbaf, Bahram Mohajer, Amir Ashraf-Ganjouei, Mahtab Mojtahed Zadeh, Ali Javinani, Hossein Sanjari Moghaddam, Mehdi Shirin Shandiz, Mohammad Hadi Aarabi

**Affiliations:** ^1^Faculty of Medicine, Tehran University of Medical Sciences, Tehran, Iran; ^2^Non-communicable Diseases Research Center, Endocrinology and Metabolism Population Sciences Institute, Tehran University of Medical Sciences, Tehran, Iran; ^3^Department of Medical Physics, Zahedan University of Medical Sciences, Zahedan, Iran

**Keywords:** Parkinson's disease, insulin-like growth factor-1, connectometry, diffusion tensor imaging, synuclein, amyloid, tau, dopamine

## Abstract

**Background:** Growing evidence shows that impaired signaling of Insulin-like Growth Factor-1 (IGF-1) is associated with neurodegenerative disorders, such as Parkinson's disease (PD). However, there is still controversy regarding its proinflammatory or neuroprotective function. In an attempt to elucidate the contribution of IGF-1 in PD, we aimed to discover the relation between serum IGF-1 levels in drug-naïve early PD patients and cerebrospinal fluid (CSF) biomarkers as well as microstructural changes in brain white matter.

**Methods:** The association between quartiles of serum IGF-1 levels and CSF biomarkers (α-synuclein, dopamine, amyloid-β_1−42_, total tau, and phosphorylated tau) was investigated using adjusted regression models in 404 drug-naïve early PD patients with only mild motor manifestations and 188 age- and sex-matched healthy controls (HC) enrolled in the Parkinson's Progression Markers Initiative (PPMI). By using region of interest analysis and connectometry approach, we tracked the white matter microstructural integrity and diffusivity patterns in a subgroup of study participants with available diffusion MRI data to investigate the association between subcomponents of neural pathways with serum IGF-1 levels.

**Results:** PD patients had higher levels of IGF-1 compared to HC, although not statistically significant (mean difference: 3.60, *P* = 0.44). However, after adjustment for possible confounders and correction for False Discovery Rate (FDR), IGF-1 was negatively correlated with CSF α-synuclein, total and phosphorylated tau levels only in PD subjects. The imaging analysis proved a significant negative correlation (FDR corrected *P*-value = 0.013) between continuous levels of serum IGF-1 in patients with PD and the connectivity, but not integrity, in following fibers while controlling for age, sex, body mass index, depressive symptoms, education years, cognitive status and disease duration: middle cerebellar peduncle, cingulum, genu and splenium of the corpus callosum. No significant association was found between brain white matter microstructral measures or CSF markers of healthy controls and levels of IGF-1.

**Conclusion:** Altered connectivity in specific white matter structures, mainly involved in cognitive and motor deterioration, in association with higher serum IGF-1 levels might propose IGF-1 as a potential associate of worse outcome in response to higher burden of α-synucleinopathy and tauopathy in PD.

## Introduction

Parkinson's disease (PD) is the second most common neurodegenerative disorder after Alzheimer's disease (AD). It is proposed that progressive damage of α-synuclein inclusions to the neural cells by causing neuroinflammation and oxidative imbalance plays the central role in PD pathology (1–4). In addition, the existence of extracellular plaques of amyloid-beta and intracellular aggregation of abnormal tau proteins, the main pathological insults in AD, are as well documented in PD with a great impact on the development of cognitive decline and dementia (5, 6). Presentation of motor and non-motor symptoms of PD may be delayed after a long preclinical phase of neuronal loss (7). Discovering biomarkers of this silent neurodegeneration has recently captured attention in early diagnosis of PD, before the emergence of the debilitating motor and cognitive symptoms, when neuroprotective measures may be promising (8). Among postulated markers, Insulin-like growth factor-1 (IGF-1) has drawn attention as a possible target of modification in the disease course (9, 10).

IGF-1 is a potent neurotrophic factor, involved in neural plasticity, differentiation, myelination, and survival (11), and is increased in the neuroinflammatory responses (12). In spite of some evidence of inhibitory effect of IGF-1 on the inflammatory cascade in a reactive cell (13), there is still controversy regarding the proinflamatory role of this factor or its protective function *in vivo*. Altered IGF-1 signaling is suggested to be associated with neurodegenerative disorders, such as PD (14, 15). Dysregulated signaling of IGF-1 involves brain tissues of post-mortem PD in several regions beyond substantia nigra, in concordance with heterogenicity of symptoms in PD (16). Increased levels of IGF-1 in serum or CSF of early PD patients have been reported in some (17–20), but not all previous studies (21, 22). However, it is not clear whether this is a chronic increase, if there is at all, with imposed susceptibility to neurodegeneration, a compensatory response against neural damage, or merely a consequence of ongoing neurodegenerative processes. However, studies on preclinical models of PD and *in vitro* cell cultures have implicated the neuroprotective effects of IGF-1 in terms of reduced apoptosis, prevention of loss of dopaminergic neurons, blocking formation of α-synuclein aggregates, rescuing neurons from amyloid plaques and neurofibrillary tangles, as well as improvement of motor deficits in the clinical aspect (23–29). These findings imply a possible therapeutic potential of the IGF-1 signaling pathway (9), beyond the proposed role of this factor as a PD biomarker.

Recently, advanced neuroimaging techniques have provided valuable data to detect early PD (8). Diffusion MRI (dMRI) is a powerful tool to identify white matter microstructural changes and has revolutionized our understanding of the neuropathology of PD. Studies have shown that Lewy bodies and coexistent AD-type pathology (amyloid plaques and neurofibrillary tangles) are associated with white matter microstructural damage in PD (30, 31). The most frequently investigated diffusivity metrics, fractional anisotropy (FA), mean diffusivity (MD), radial diffusivity (RD), and axial diffusivity (AD) are indirect indicators of almost every pathology affecting the white matter (32) and have yielded valuable information about the microstructural changes in PD and its clinical relevance (33). dMRI connectomery is a novel analytical method with higher sensitivity and specificity than conventional diffusion metrics, as it only probes subcomponents of the neural tracts, which are significantly related to the study variable and has bypassed the limitations of end-to-end fiber tracking methods (34). In addition, Connectometry is depended on the Spin Distribution Function (SDF), which measures the density of water diffusion in any direction and reports peak SDF for each direction as quantitative anisotropy (QA). Thus, it is highly efficient to measure the connectivity between adjacent voxels of a neural tract and results in what is called “local connectome fingerprint,” as it is highly specific to each individual (35).

To date, few studies have explored IGF-1 associations in PD, and no study has yet investigated the association between IGF-1 levels and microstructural white matter disruptions in PD patients. In order to understand the role of IGF-1 in PD pathophysiology, we investigated its association with CSF biomarkers and white matter microstructural changes in early drug-naïve patients with PD. We probed the integrity and connectivity of fiber tracts in relation to serum IGF-1 levels by performing atlas-based region of interest and connectometry analyses of diffusion MRI data.

## Patients and methods

### Participants

Data used in this study were obtained from the Parkinson's Progression Markers Initiative (PPMI) database (36) (www.ppmi-info.org/data). The study was approved by the institutional review board of all participating sites. Written informed consent was obtained from all participants before study enrolment. The study was performed in accordance with relevant guidelines and regulations according to the PPMI protocol. The participants were tested and confirmed negative for any neurological disorders apart from PD. Inclusion of PD patients in PPMI meets the following criteria: (1) patients should have at least two of the following signs and symptoms: resting tremor, bradykinesia, and rigidity or either asymmetric resting tremor or asymmetric bradykinesia; (2) maximum of 2 years passed from PD diagnosis in the screening stage; (3) baseline H & Y stage of I or II; (4) Loss of dopaminergic neurons confirmed on DAT scans; (5) no use of PD medications within at least 6 months prior to the baseline visit; and (6) aged ≥30 years at the time of diagnosis. Only PD patients and age- and sex-matched healthy control subjects (HC), recruited from 11 different centers, for whom baseline visit values of covariates of interest, i.e., CSF biomarkers, and serum IGF-1 levels were available were enrolled in this study. Among these participants, subjects who had baseline dMRI data with acceptable quality (imaging subgroup) were included in the subsequent imaging analyses.

Baseline assessment of global cognitive status was evaluated using Montreal Cognitive Assessment (MoCA). Data of the 15-item geriatric depression scale (GDS) were used to evaluate depressive symptoms in the study groups. Motor symptom severity was scored based on the Movement Disorders Society Unified Parkinson's Disease Rating Scale (MDS-UPDRS) part III. A detailed method of each can be found on the PPMI website (http://www.ppmi-info.org/study-design/research-documents-and-sops/).

### CSF biomakers measurement

At the baseline visit, CSF samples were obtained from PPMI participants using lumbar puncture at each participating site. Concentrations of α-synuclein, amyloid-beta (Aβ_1−42_), total tau (*T*-tau), phosphorylated tau (*P*-tau), and Dopamine were measured by a commercially available enzyme-linked immunosorbent assay kit (Covance). The detailed method can be found on the PPMI website (http://www.ppmi-info.org/study-design/research-documents-and-sops/).

### IGF-1 measurement

As outlined in the PPMI laboratory manual, venous blood samples of participants were collected at baseline visit in the morning after an overnight fasting period. Serum IGF-1 measurement was performed using the Quantikine Human IGF-1 Immunoassay, which employs the quantitative sandwich enzyme immunoassay technique. This assay recognizes natural and recombinant human IGF1 and is calibrated against a highly purified *E. coli*-expressed recombinant human IGF1, produced at R&D Systems. Pretreatment of serum with acid-ethanol extraction was done to release IGF-1 from IGF-binding proteins. Standards and pretreated samples were pipetted into the wells on a microplate, previously coated with a monoclonal antibody specific for IGF1. After washing away unbound substances with the antibody, an enzyme-linked polyclonal antibody specific for IGF1 was added to the wells. Following a second wash, a substrate solution was added to the wells and color developed in proportion to the amount of IGF1 bound in the initial step. A microplate reader (Victor X4-Perkin Elmer) set to 450 nm was used to measure the intensity of the color. A standard curve was generated for each set of assayed samples. Intra and inter-assay precision were confirmed with multiple tests of three samples of known concentrations. Results of natural human IGF1 showed linear curves that were parallel to the standard curves obtained, using the Quantikine kit standards. The NIBSC/WHO IGF1 International Reference Reagent 02/254 was evaluated in this kit. The dose-response curve of the International Reference Reagent parallels the Quantikine standard curve. Sample values obtained with the Quantikine Human IGF1 kit were finally converted to NIBSC/WHO 02/254 values.

### MRI data acquisition

This dataset was acquired on 3 Tesla Siemens scanners, producing 64 directions of dMRI (repetition time = 7,748 MS, echo time = 86 ms; voxel size: 2.0 × 2.0 × 2.0 mm^3^; field of view = 224 × 224 mm) at b = 1,000 s/mm^2^ and one b0 image. Details of the MRI acquisition can be found in the MRI technical operations manual at (http://www.ppmi-info.org/study-design/research-documents-and-sops/).

### Diffusion MRI data analysis

Diffusion MRI data were corrected for subject motion, eddy current distortions, and susceptible artifacts due to the magnetic field inhomogeneity using ExploreDTI toolbox (37). To define the regions of interest (ROI), we performed automated atlas-based analysis with the Johns Hopkins University's Mori white matter atlas using affine and elastic registration based on “elastix”. After these preprocessing steps, FA, MD, RD, and AD values were calculated in the 48 brain regions that are provided by the Mori atlas.

Connectometry analyses (34) were performed on subgroups of PD and HC subjects separately. The diffusion data were reconstructed in the MNI space using q-space diffeomorphic reconstruction to obtain the spin distribution function (SDF) (38). Next, dMRI connectometry was used to study the association of white matter connectivity with levels of serum IGF-1. SDF was normalized. A *T*-score threshold of 2 was assigned to select local connectomes, and the local connectomes were tracked using a deterministic fiber tracking algorithm. A length threshold of 40 mm was used to select tracks. The seeding density was 50 seeds per mm^3^. Multiple regression models were used to investigate the correlation of IGF-1 with white matter quantitative anisotropy (QA), separately in PD and HC subjects, adjusted for age, sex, Body Mass Index (BMI), disease duration, and education years as covariates in the model. To estimate the False Discovery Rate (FDR), a total of 2,000 randomized permutation was applied to the group label to obtain the null distribution of the track length. The analysis was conducted using publicly available software DSI Studio (http://dsi-studio.labsolver.org/) released on 5th April 2018.

### Statistical analysis

Demographic, clinical and CSF biomarker data were compared between HC and PD groups using independent sample *t*-test and chi-square tests, and in case of non-normal distribution assessed by Shapiro-Wilk test, Kruskal-Wallis was used in a substitute. IGF-1 serum level was further compared between groups using a linear regression model, considering age, gender, and BMI as possible confounders. Statistical power was calculated for effect size = 0.1 and significance threshold = 0.05. Because of non-linear association of IGF-1 levels with dependent variables, IGF-1 values were categorized in four quartiles as follows: first quartile ≤97.9 ng/ml; second quartile = 97.9001–124 ng/ml; third quartile = 124.0001–162.200 ng/ml; and forth quartile ≥162.2001 ng/ml. Adjusted multiple regression models were used to evaluate the association of serum IGF-1 levels with CSF biomarkers and brain white matter microstructural integrity. Models were fitted separately for PD and HC subjects considering age, gender, body mass index (BMI), education years, Hoehn & Yahr (H&Y) stage, and duration of disease in months as covariates (two latter were only fitted in the model of PD subjects). Models regarding CSF biomarkers were additionally adjusted for MOCA score while excluding education years. FDR approach with the significant level of 0.05 was used to correct results for family wise error. In order to clarify the possible effect of gender, motor stage, and global cognitive status (intact cognition and mild cognitive impairment), potential effect modifications by gender, H&Y stage, and MoCA scores were examined by adding interaction terms of (IGF-1 × gender) or (IGF-1 × H&Y) or (IGF-1 × MoCA) separately to the fully adjusted models. Since *P-*values were not <0.10, no further stratification was applied based on gender, H&Y or MoCA. All the statistical analyses were done in the R statistical package (v3.4.3).

## Results

The study group consisted of 404 PD patients (263 females, mean age: 61.6 ± 9.7 years) and 188 HC (120 females, mean age: 60.8 ± 11.3 years). Table 1 shows group-specific demographics and baseline level characteristics of total and imaging subgroup participants. All PD subjects had disease duration <36 months with a mean duration of 6.6 ± 6.6 months. 180 patients were at H&Y stage 1 compatible with unilateral motor involvement and mild to no disability. Two patients were in stage 3, and the remaining patients were in stage 2 of PD progression with bilateral involvement but no impairment in their balance. HC subjects were all cognitively intact according to the MoCA scale (mean MoCA score: 28.23 ± 1.11). 88 PD subjects had the score between 19 and 25 compatible with mild cognitive impairment, and 2 had the score below 19 categorized as severe impairment. 256 PD and 131 HC participants scored 5 to 8 in geriatric depression score indicative of mild depression while the remaining subjects scored below 4. Mean BMI of the PD participants was 27.12 ± 4.52. As the effect of BMI on the IGF-1 level was controlled in multiple regression analysis, we did not exclude obese participants. Of note, there were few PD patients with comorbid disorders, such as diabetes mellitus (17), thyroid dysfunction (59), and cancer (11). Four patients were taking corticosteroids at the baseline visit. However, excluding these participants did not change the results.

**Table 1:** Demographic and baseline clinical data of participants.

	**Total population**			**Imaging population**		
**Groups**	**HC**	**PD**	***P*****-value**	**HC**	**PD**	***P*****-value**
	**(188)**	**(404)**		**(57)**	**(82)**	
Age at baseline visit in years, mean (SD)	60.87 (11.30)	61.66 (9.74)	0.61	59.57 (10.76)	57.70 (8.89)	0.173
Sex, Female, no. (%)	120 (63.8)	263 (65.1)	0.835	38 (66.7)	51 (62.2)	0.718
BMI (Kg/m^2^), mean (SD)	26.97 (4.41)	27.12 (4.52)	0.756	26.66 (4.42)	26.84 (4.45)	0.984
Education years, mean (SD)	16.08 (2.89)	15.57 (2.99)	0.061	15.40 (2.84)	15.21 (2.81)	0.795
IGF-1 (ng/Ml), mean (SD)	134.55 (56.33)	136.79 (54.42)	0.444	132.09 (55.64)	136.26 (46.06)	0.281
Adjusted IGF-1 difference^*^	Mean difference: 3.60		0.444	Mean difference: 3.16		0.714
	Power = 1			Power = 0.96		
IGF-1 quartiles (%)			0.013			0.518
1	39 (20.7)	109 (27.0)		16 (28.1)	20 (24.4)	
2	62 (33.0)	87 (21.5)		14 (24.6)	14 (17.1)	
3	48 (25.5)	99 (24.5)		15 (26.3)	23 (28.0)	
4	39 (20.7)	109 (27.0)		12 (21.1)	25 (30.5)	
Adjusted IGF-1 quartiles difference^*^	Estimate: 0.11		0.487	Estimate: 0.34		0.280
	Power = 1			Power = 0.97		
Duration of disease in months, mean (SD)		6.66 (6.66)			6.76 (6.99)	
H & Y stage no. (%)						
0	188 (100.0)	0 (0.0)		57 (100.0)	0 (0.0)	
1	–	180 (44.6)		–	37 (45.1)	
2	–	222 (55.0)		–	45 (54.9)	
3	–	2 (0.5)		–	0 (0.0)	
UPDRS III, mean (SD)	1.16 (2.15)	20.72 (8.80)	<0.001	0.72 (1.45)	20.52 (8.76)	<0.001
GDS score, mean (SD)	4.74 (1.00)	4.54 (1.11)	0.052	4.54 (1.02)	4.38 (1.28)	0.647
MOCA score, mean (SD)	28.23 (1.11)	27.10 (2.34)	<0.001	28.19 (1.16)	27.77 (1.90)	0.46
Aβ_1−42_, mean (SD)	1,024.18 (506.01)	914.94 (414.00)	0.028	973.55 (485.41)	906.41 (355.65)	0.728
Phosphorylated Tau, mean (SD)	16.86 (8.57)	14.36 (5.46)	0.001	15.28 (6.79)	13.90 (4.43)	0.561
Total Tau, mean (SD)	190.29 (81.35)	167.73 (58.63)	0.004	175.15 (72.17)	163.68 (48.81)	0.717
α-synuclein, mean (SD)	1,707.07 (760.51)	1,520.66 (673.34)	0.003	1,484.31 (598.53)	1,519.03 (637.42)	0.689
Dopamine, mean (SD)	0.01 (0.01)	0.01 (0.01)	0.021	0.01 (0.00)	0.01 (0.01)	0.102

H & Y stage, Hoen and Yahr stage; BMI, body mass index; IGF, insulin-like growth factor; UPDRS III, unified Parkinson's disease rating scale part III; GDS, geriatric depression scale; MoCA, Montreal Cognitive Assessment. Shapiro–Wilk test used to assess normality of distribution. For the normally distributed continous variables, t-test was implemented and in the case of non-normality in distribution Kruskal-Wallis test was used. Categorical variables were assessed using Pearson Chi-square and fisher exact test.

* *Adjusted difference in IGF-1 serum levels were assessed using multiple regression model considering age, gender, and BMI as covariates. Power calculated estimating effect size = 0.1 and significance level = 0.05*.

There was no significant difference between PD and HC group regarding age, sex ratio, BMI, and years of education. However, PD subjects had significantly lower scores in MoCA (*p* < 0.001) and marginally higher scores of GDS (*P*: 0.052). All PD patients had significantly lower Aβ_1−42_, *T*-tau, *P*-tau, α-synuclein, and dopamine compared to HC.

In the subgroup of participants included in the imaging analysis, there were no differences in age, gender ratio, BMI, education, as well as clinical and CSF biomarkers, except for UPDRS III, between 82 PD patients and 57 HC. All PD subjects of the imaging population had an H&Y score of 1 or 2 and none of them scored below 19 on the MoCA.

CSF concentration of IGF-1 was not statistically different between PD and HC subjects before and after adjustment (PD: 136.79 ± 54.42, HC: 134.55 ± 56.33 ng/ml). Distribution of serum IGF-1 level quartiles were different between diagnoses, but the result did not remain statistically significant after adjustment for age, gender, and BMI (mean difference: 3.60, *P*: 0.4, estimated power ≈ 1).

### Association of serum IGF-1 level quartiles with CSF biomarkers

**Figure 1:**
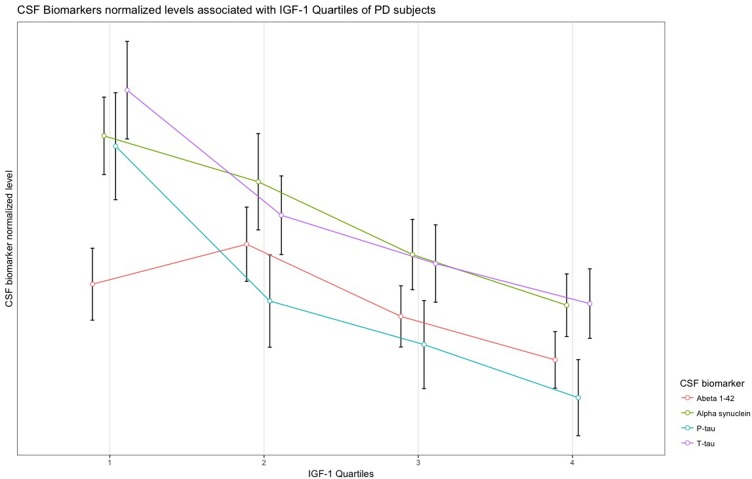
Normalized levels of the CSF biomarkers associated with IGF-1 quartiles in patients with PD.

In PD subjects, a significant negative association was found between serum IGF-1 quartiles and CSF α-synuclein, *T*-tau, and *P*-tau concentrations. Results remained significant after adjustment and FDR correction of *p*-values. The remaining associations between IGF-1 quartiles and CSF biomarkers of PD and HC subjects were not statistically meaningful (Table 2). Scaled (0–100) levels of CSF biomarker through IGF-1 quartiles are illustrated in the Figure 1.

**Table 2:** Association of CSF biomarkers with IGF-1 quartiles in PD and HC subjects.

**IGF-1**	**1st quartile**	**2nd quartile**	**3rd quartile**	**4th quartile**	**Estimate ± SE, *P*-value**
PD	109 subjects	87 subjects	99 subjects	109 subjects	
Dopamine	0.01 (0.01)	0.01 (0.01)	0.01 (0.01)	0.01 (0.01)	0 ± 0.001, *P*: 0.788
α-synuclein	1,669.76 (703.08)	1,588.89 (782.35)	1,460.56 (607.11)	1,371.28 (568.47)	−179.64 ± 66.78, *P*: 0.007^*^
Total Tau (*t*-Tau)	185.72 (71.02)	168.03 (51.26)	161.21 (53.56)	155.54 (50.80)	−15.8 ± 5.705, *P*: 0.006^*^
Phosphorylated Tau (*p*-Tau)	16.12 (6.46)	14.30 (5.00)	13.79 (5.07)	13.17 (4.62)	−1.58 ± 0.53, *P*: 0.003^*^
Aβ_1−42_	940.37 (466.87)	991.03 (428.14)	899.54 (375.93)	844.48 (370.57)	−82.41 ± 41.90, *P*: 0.05
*t*-Tau Aβ_1−42_ ratio	0.22 (0.11)	0.18 (0.06)	0.20 (0.09)	0.21 (0.11)	0.003 ± 0.01, *P*: 0.718
*p*-Tau Aβ_1−42_ ratio	0.02 (0.01)	0.02 (0.01)	0.02 (0.01)	0.02 (0.01)	0 ± 0.001, *P*: 0.899
*p*-Tau *t*-Tau ratio	0.09 (0.01)	0.08 (0.01)	0.09 (0.01)	0.08 (0.01)	−0.002 ± 0.001, *P*: 0.043
HC	39 subjects	62 subjects	48 subjects	39 subjects	
Dopamine	0.01 (0.00)	0.01 (0.01)	0.01 (0.00)	0.01 (0.00)	−0.002 ± 0.002, *P*: 0.329
α-synuclein	1,895.98 (926.65)	1,773.07 (690.23)	1,689.51 (737.16)	1,451.42 (675.67)	−224.88 ± 125.87, *P*: 0.076
Total Tau (*t*-Tau)	211.65 (113.03)	198.03 (73.56)	181.43 (66.66)	168.86 (69.07)	−17.68 ± 13.12, *P*: 0.18
Phosphorylated Tau (*p*-Tau)	19.46 (13.83)	17.55 (7.16)	15.80 (6.05)	14.62 (5.88)	−2.17 ± 1.39, *P*: 0.12
Aβ_1−42_	947.08 (372.86)	1,080.15 (549.11)	1,070.43 (445.11)	958.47 (602.31)	17.83 ± 86.48, *P*: 0.837
*t*-Tau Aβ_1−42_ ratio	0.26 (0.25)	0.21 (0.12)	0.18 (0.07)	0.21 (0.13)	−0.02 ± 0.025, *P*: 0.411
*p*-Tau Aβ_1−42_ ratio	0.03 (0.03)	0.02 (0.01)	0.02 (0.01)	0.02 (0.01)	−0.003 ± 0.003, *P*: 0.382
*p*-Tau *t*-Tau ratio	0.09 (0.01)	0.09 (0.01)	0.09 (0.01)	0.09 (0.01)	0 ± 0.001, *P*: 0.814

Values are reported as mean (SD).

* *Significant after FDR (threshold < 0.05)*.

### Association of serum IGF-1 level with white matter microstructural integrity and connectivity

No association was found between IGF-1 levels, neither continuous nor quartile, with FA, MD, RD, or AD values of whole brain white matter tracts in PD or HC (Supplementary Table). There was also no interaction between IGF-1 levels and diagnosis with regional FA and MD values.

Results from multiple regression models in dMRI connectometry of PD patients revealed areas where white matter QA was negatively correlated with IGF-1 level. More specifically, the connectometry analysis identified lower connectivity in middle cerebellar peduncle (MCP), left and right cingulum, and genu and splenium of the corpus callosum (CC) in correlation with serum IGF-1 continuous level after adjustment for possible confounders and FDR correction of *p*-values (FDR = 0.013). Dividing patients based on IGF-1 quartiles, connectometry analysis revealed a negative association between IGF-1 continuous levels in the first quartile and QA of aforementioned tracts (FDR: 0.04). Bilateral cingulum and bilateral corticospinal tracts (CST) were the only areas with a trend to significantly lower connectivity in terms of lower QA in association with higher IGF-1 continuous levels in the fourth quartile (FDR: 0.05). No association was observed between IGF-1 continuous levels in the second or third quartiles and white matter QA in PD. When considering IGF-1 quartiles as ordinal variable, MCP, bilateral fornix, cingulum, genu and body of the CC showed significantly negative association in PD, while splenium and bilateral corticospinal tracts were positively correlated with IGF-1 quartiles with *P-*values just below the significant threshold (FDR: 0.033 and 0.049, respectively).

No significant association was found between IGF-1 levels and white matter QA in all HC or divided based on IGF-1 quartiles.

## Discussion

In the present study, we have investigated the association of serum IGF-1 levels with CSF biomarkers, as well as whole brain white matter microstructural connectivity and integrity to elucidate the possible role of serum IGF-1 in the central pathophysiology of PD. PD type and AD-type CSF biomarkers in our subjects were lower compared to HC, in agreement with previous studies (39–42). It is speculated that precipitation of α-synuclein, amyloid- β, or tau proteins lowers their free concentration in the CSF. Despite non-significant differences between serum IGF-1 levels of early drug-naïve PD patients and HC, we could reveal that decreased concentration of CSF α-synuclein and tau proteins correlate with higher serum IGF-1 quartiles in PD patients, a finding that did not replicate in HC. In addition, we found that structural brain dysconnectivity, but not disintegrity in cingulum, genu, and MCP is associated with higher serum IGF-1 continuous levels only in PD patients, and more particularly in the subgroup of patients with serum IGF-1 levels in the first quartile. On the other hand, higher IGF-1 quartiles correlated with higher connectivity in the splenium and CST, and lower connectivity in the cingulum, fornix, and genu and body of the CC.

IGF-1 receptors are distributed throughout the brain, with high densities in the hippocampus, frontal cortex, and cerebellum (43, 44). The cingulum bundle and CC have pivotal roles in cognitive function. The most anterior parts of these structures contribute to goal-directed behavior and attention modulation through the network of prefrontal projections, while the most posterior parts are involved in regulating memory with their harbored temporal and parahippocampal connections (45–47). Remarkably, the cingulum and CC are the fibers most affected in the mild cognitive impairment, AD, and PD with dementia; The microstructural compromise in the parahippocampal and posterior cingulum, and the splenium of the CC is replicated in several studies of amnesic mild cognitive impairment and AD (48–52). On the other hand, normal aging is shown to be more confined to the frontal circuit with the coexistence of disruptions in the genu and anterior cingulum (53). There are also several reports of the centrality of lower integrity of CC and cingulum in relation with poorer cognition and transition to demented state in patients with PD (54–58). Of note, the prefrontal white matter is the most consistently described region in PD (33), considering the executive dysfunction as the main cognitive disturbance in this neurodegenerative disorder (59). However, the involvement of posterior cognitive circuits confers an increased risk of dementia in PD (60). Moreover, The spread of α-synuclein aggregations to the limbic and neocortical structures can be used to differentiate between PD with and without dementia (61). In addition, AD-type pathology has synergistic pathology with a higher burden of α-synucleinopathy in the limbic and cortical structures of demented patients with PD (62).

Previously, Picillo et al. investigated the association between the serum IGF-1 quartiles with performance in several aspects of cognition in a similar group of subjects enrolled in PPMI database (22). They demonstrated that the lowest serum IGF-1 level is associated with cognitive decline in the domains of executive function, attention and verbal memory, which are mainly mediated by the prefrontal and temporal regions. The association of lower serum IGF-1 levels with poor cognitive ability is replicated in other studies as well (22, 63, 64). Furthermore, it is demonstrated that IGF-1 level rises non-significantly early in the course of PD, peaks at H&Y stage 2 and then decreases when PD ends in stages 3–5 (21, 65). Also, Godau et al. found that higher serum IGF-1 levels are correlated with shorter disease duration (17). A dome-shaped pattern through disease course on the side of *in vitro* and *in vivo* neuroprotective roles suggested for IGF-1 (18, 66, 67), along with our imaging findings of lower white matter QA and CSF biomarkers associated with higher IGF-1 in early PD, may propose a primary compensatory rise in IGF-1 signaling against neural damage of Lewy bodies and neurofibrillary tangles, with consequent depletion of neuroprotective capacity later in the course of the disease (17, 65). However, the exact pathophysiology still needs to be elucidated.

Studies have demonstrated conflicting results trying to link the level of serum IGF-1 to motor severity in PD. Picillo et al. have surveyed the relationship between serum IGF-1 level and progression of motor dysfunction in a 2-years follow-up of 37 early PD patients. They found that higher IGF-1 at baseline predicts more severe motor impairment with possibly better response to dopaminergic administration (20). A negative association was also replicated in a cohort of 79 Japanese PD patients. However, subjects were mostly in H&Y stages 3 and more, and the association was not confirmed in the subgroup of early drug naïve PD patients (65). Godau et al. showed a correlation between decreased serum IGF-1 levels and severity of motor symptoms among 15 newly diagnosed PD patients, but a positive correlation in 11 persons with suspected dopaminergic deficit and putatively at risk for PD (19). Considering limited data on CSF dopamine assessment in PPMI database (total of 90 PD subjects in our sample), we did not find any association between IGF-1 quartiles and dopamine in early drug-naïve PD. However, there was a negative correlation between IGF-1 level quartiles and the CSF concentrations of α-synuclein and tau proteins. Although dopaminergic depletion is considered the mainstay of extrapyramidal motor manifestations in PD, Kang et al. have shown that CSF α-synuclein and *T*-tau are decreased in early PD patients, and this reduction is associated with severity of motor symptoms (41, 42). They also revealed that patients with lower *P*-tau are more likely to have the non-tremor dominant phenotype, who more often experience cognitive deterioration (68). Postural instability, bradykinesia or freezing of gait are shown to be related to structural incoherence in the genu, body and splenium of the CC, and cingulum (69–71). Impaired integrity in MCP is also demonstrated in PD patients with freezing of gait (72–74). Besides associations with motor manifestations, structural disturbance of the cerebellum is also linked to cognitive and executive dysfunction (75–77). Considering observed disconnectivity in the cingulum, CC and MCP in relation to higher IGF-1 continous levels, increased IGF-1 in early PD may alarm the development of severe motor symptoms in the course of the disease. To further complicate this senario, we observed a positive association between IGF-1 quartiles and the connectivity in the splenium and CST. There is multitude evidence concerning the reputation of incoherence in these structures regarding motor and cognitive deterioration in PD (33). The positive association of IGF-1 quartile specifically with these structures may reinforce the debate on the compensatory role of IGF-1 in early phases of PD. Although it seems that this compensation does not completely overcome the ongoing neural damage, as noted by its negaitive association with other major white matter pathways. To support this notion, a comprehensive *in vivo* and *in vitro* preclinical study has demonstrated that IGF-1 downstream signaling pathways enhance the axonal outgrowth in the CST (78). Another study on the mouse model of CC demyelination has revealed that IGF-1 expression is upregulated in the process of neural repair (79). Still, studies on human subjects with longitudinal design are needed to confirm this hypothesis.

Although the major white matter tracts with a central role in cognitive impairment and dementia, and with fundamental associations with motor severity showed altered connectivity in correlation with serum IGF-1 levels, there was no such association with white matter FA, MD, RD, or AD values. In fact, tensor measures rely on the diffusivity of each voxel and mainly detect the pathology in fiber tracts, while connectivity metrics, like QA, are based on density for each fiber orientation and count for individual differences (35). Therefore, QA has resolved the shortage of FA in the areas of crossing fibers. Notably, we were able to detect the microstructural changes in short association fibers, which are located in the region with high crossing fibers, in patients with PD by using the connectometry approach (80). Furthermore, several pathological features, such as axonal loss, demyelination, inflammation, or edema can alter FA. Edema without demyelination may increase FA, and alterations of FA may take a long time to appear in the setting of gradual neuronal loss. However, QA is more related to the neural changes and less affected by the inflammation and edema, and less susceptible to partial volume effect (81). Therefore, connectivity is more sensitive and specific that tensor analyses. Longer duration of the disease might probably reveal FA changes in specific white matter pathways in relation to IGF-1, a hypothesis that needs to be investigated in well-designed cohort studies. Studying PD patients in advanced stages with dementia may further clarify the possible role of IGF-1 signaling in specific white matter disruptions.

The large sample size, both in total included population and imaging subgroup, has devoted a great statistical power to the observed results. Considering the previously described higher levels of IGF-1 in early phases of neurodegenerative disorders, studying early stages of PD without the conferring effects of treatment appear the best point in the disease course to evaluate the possible role of IGF-1 in the central pathophysiology of PD. Nevertheless, despite growing evidence of IGF-1 protection against amyloid accumulation or dopamine depletion, we could not find any significant association between IGF-1 levels and CSF concentrations of dopamine or Aβ_1−42_. In addition, follow-up studies are warranted to clarify the generalizability of these results to more advanced stages and to determine the casual relationship between this promising neurotrophic factor and progression of PD. Even though we have considered major possible confounders and corrected our results for multiple comparisons, high prevalence of mild depression in the study population, lower number of subjects provided with dopamine levels and measurement of IGF-1 levels in the serum and not CSF should be kept in mind while interpreting our results.

## Conclusion

In this study, we showed that despite no difference between serum IGF-1 level in PD compared to HC, it is negatively associated with CSF *T*-tau, *P*-tau, and α-synuclein, as well as altered microstructural connectivity in the several brain white matter regions of early drug naïve PD patients with mild motor symptoms. We demonstrated that lower connectivity measures in MCP, cingulum, and CC are correlated with higher continuous levels of IGF-1 exclusively in PD subjects. Disruption of these pathways is previously described in PD and in association with debilitating motor and cognitive disturbances. It is not clear whether the correlation of IGF-1 levels with alteration of CSF biomarkers and neural damage is a modulation in its signaling pathway as a compensatory mechanism, or is only a parallel finding to disease progression. In addition, we found higher IGF-1 quartiles in association with higher connectivity in the splenium and bilateral CST. This specific result may reinforce the debate on the compensatory role of IGF-1 against the ongoing neural damage in PD at least in some specific brain pathways. More studies are needed to assess these findings in follow-up visits and inspect the development of motor or cognitive symptoms related to the changes in the CSF biomarkers and microstructural alterations in the specified pathways.

## Ethics statement

All procedures performed here, including human participants were in accordance with the ethical standards of the institutional research committee and with the 1964 Helsinki declaration and its later amendments or comparable ethical standards.

## Author contributions

MA conceived of the presented idea. FG, BM, and MA contributed to the design and implementation of the research. MA and BM performed the analytic calculations. AJ and AA-G drafted the manuscript. FG wrote the manuscript with input from all authors. HS, MM, and MS contributed to data collection, aided in interpreting the results, and worked on the manuscript. All authors provided critical feedback and helped shape the research, analysis, and manuscript.

### Conflict of interest statement

The authors declare that the research was conducted in the absence of any commercial or financial relationships that could be construed as a potential conflict of interest.
