# Supplementary material for: Serum Insulin-Like Growth Factor-1 in Parkinson's Disease; Study of Cerebrospinal Fluid Biomarkers and White Matter Microstructure
Source: Front Endocrinol (Lausanne). 2018 Nov 2;9:608. doi: 10.3389/fendo.2018.00608 (PMC6224341; doi:10.3389/fendo.2018.00608)
Supplement: Supplementary file 1 [file Table_1.DOCX]

| IGF-1 quartiles | | 1 | 2 | 3 | 4 |  |  |
| --- | --- | --- | --- | --- | --- | --- | --- |
| Count | | 33 | 22 | 28 | 24 | Difference (CI) | FDR P value |
|  | FA | Mean (SD) | Mean (SD) | Mean (SD) | Mean (SD) |  |  |
| Anterior corona radiata left | | 0.38 (0.03) | 0.37 (0.04) | 0.38 (0.03) | 0.37 (0.04) | -0.006 (-0.018 - 0.005) | 0.582 |
| Anterior corona radiata right | | 0.37 (0.03) | 0.36 (0.04) | 0.37 (0.03) | 0.36 (0.04) | -0.008 (-0.02 - 0.004) | 0.582 |
| Anterior limb of internal capsule left | | 0.50 (0.04) | 0.50 (0.04) | 0.50 (0.05) | 0.49 (0.05) | -0.012 (-0.03 - 0.007) | 0.582 |
| Anterior limb of internal capsule right | | 0.51 (0.05) | 0.50 (0.05) | 0.51 (0.05) | 0.51 (0.05) | -0.007 (-0.025 - 0.011) | 0.74 |
| Body of corpus callosum left | | 0.52 (0.06) | 0.48 (0.07) | 0.51 (0.07) | 0.50 (0.07) | -0.009 (-0.034 - 0.016) | 0.781 |
| Cerebral peduncle left | | 0.52 (0.05) | 0.52 (0.05) | 0.52 (0.08) | 0.51 (0.06) | -0.014 (-0.038 - 0.009) | 0.582 |
| Cerebral peduncle right | | 0.54 (0.05) | 0.54 (0.05) | 0.53 (0.07) | 0.52 (0.07) | -0.024 (-0.047 - 0) | 0.384 |
| Cingulum (Caudal anterior) left | | 0.38 (0.06) | 0.35 (0.08) | 0.38 (0.07) | 0.37 (0.06) | -0.006 (-0.032 - 0.02) | 0.892 |
| Cingulum (Caudal anterior) right | | 0.41 (0.07) | 0.38 (0.09) | 0.42 (0.07) | 0.41 (0.07) | 0.005 (-0.023 - 0.032) | 0.892 |
| Cingulum (hippocampus) left | | 0.31 (0.04) | 0.32 (0.04) | 0.30 (0.05) | 0.32 (0.03) | -0.004 (-0.019 - 0.011) | 0.863 |
| Cingulum (hippocampus) right | | 0.31 (0.05) | 0.33 (0.04) | 0.29 (0.05) | 0.31 (0.04) | -0.008 (-0.027 - 0.01) | 0.653 |
| Corticospinal tract left | | 0.39 (0.04) | 0.39 (0.05) | 0.38 (0.07) | 0.38 (0.08) | -0.01 (-0.034 - 0.014) | 0.684 |
| Corticospinal tract right | | 0.41 (0.05) | 0.41 (0.06) | 0.39 (0.08) | 0.40 (0.08) | -0.016 (-0.042 - 0.01) | 0.582 |
| External capsule left | | 0.38 (0.03) | 0.39 (0.03) | 0.37 (0.03) | 0.37 (0.04) | -0.01 (-0.023 - 0.003) | 0.538 |
| External capsule right | | 0.40 (0.04) | 0.41 (0.03) | 0.40 (0.04) | 0.40 (0.04) | -0.009 (-0.025 - 0.006) | 0.582 |
| Fornix (column and body of fornix) left | | 0.33 (0.09) | 0.32 (0.08) | 0.33 (0.10) | 0.35 (0.10) | 0.016 (-0.013 - 0.044) | 0.582 |
| Fornix (cres) / Stria terminalis (can not be resolved with current resolution) left | | 0.31 (0.06) | 0.30 (0.04) | 0.31 (0.07) | 0.31 (0.04) | 0 (-0.018 - 0.018) | 0.993 |
| Fornix (cres) / Stria terminalis (can not be resolved with current resolution) right | | 0.37 (0.06) | 0.37 (0.05) | 0.37 (0.08) | 0.37 (0.05) | -0.004 (-0.026 - 0.019) | 0.892 |
| Genu of corpus callosum left | | 0.48 (0.06) | 0.46 (0.06) | 0.48 (0.08) | 0.48 (0.05) | 0 (-0.024 - 0.023) | 0.993 |
| Inferior cerebellar peduncle left | | 0.41 (0.03) | 0.42 (0.03) | 0.41 (0.06) | 0.41 (0.07) | -0.012 (-0.032 - 0.009) | 0.582 |
| Inferior cerebellar peduncle right | | 0.42 (0.03) | 0.42 (0.03) | 0.40 (0.06) | 0.40 (0.06) | -0.021 (-0.04 - -0.002) | 0.384 |
| Medial lemniscus left | | 0.49 (0.03) | 0.49 (0.03) | 0.47 (0.09) | 0.48 (0.07) | -0.016 (-0.04 - 0.009) | 0.582 |
| Medial lemniscus right | | 0.49 (0.04) | 0.49 (0.03) | 0.48 (0.08) | 0.46 (0.09) | -0.028 (-0.054 - -0.002) | 0.384 |
| Middle cerebellar peduncle | | 0.40 (0.03) | 0.40 (0.03) | 0.38 (0.06) | 0.38 (0.05) | -0.018 (-0.036 - -0.001) | 0.384 |
| Pontine crossing tract (a part of MCP) | | 0.43 (0.03) | 0.43 (0.04) | 0.40 (0.07) | 0.40 (0.06) | -0.027 (-0.048 - -0.006) | 0.336 |
| Posterior corona radiata left | | 0.42 (0.03) | 0.42 (0.03) | 0.42 (0.03) | 0.42 (0.04) | -0.007 (-0.018 - 0.005) | 0.582 |
| Posterior corona radiata right | | 0.41 (0.03) | 0.42 (0.03) | 0.41 (0.03) | 0.40 (0.04) | -0.011 (-0.021 - 0) | 0.384 |
| Posterior limb of internal capsule left | | 0.59 (0.03) | 0.60 (0.03) | 0.59 (0.05) | 0.59 (0.05) | -0.014 (-0.031 - 0.003) | 0.502 |
| Posterior limb of internal capsule right | | 0.62 (0.03) | 0.63 (0.03) | 0.62 (0.05) | 0.62 (0.05) | -0.008 (-0.024 - 0.008) | 0.599 |
| Posterior thalamic radiation (include optic radiation) left | | 0.45 (0.04) | 0.44 (0.03) | 0.44 (0.05) | 0.44 (0.05) | -0.009 (-0.026 - 0.008) | 0.582 |
| Posterior thalamic radiation (include optic radiation) right | | 0.44 (0.03) | 0.44 (0.03) | 0.44 (0.05) | 0.43 (0.04) | -0.009 (-0.025 - 0.006) | 0.582 |
| Retrolenticular part of internal capsule left | | 0.52 (0.04) | 0.52 (0.04) | 0.51 (0.05) | 0.51 (0.06) | -0.023 (-0.04 - -0.006) | 0.32 |
| Retrolenticular part of internal capsule right | | 0.58 (0.04) | 0.57 (0.03) | 0.56 (0.05) | 0.56 (0.05) | -0.023 (-0.04 - -0.006) | 0.32 |
| Sagittal stratum (include inferior longitidinal fasciculus and inferior fronto-occipital fasciculus) left | | 0.43 (0.04) | 0.42 (0.03) | 0.41 (0.06) | 0.42 (0.05) | -0.014 (-0.032 - 0.004) | 0.538 |
| Sagittal stratum (include inferior longitidinal fasciculus and inferior fronto-occipital fasciculus) right | | 0.45 (0.04) | 0.44 (0.03) | 0.44 (0.05) | 0.44 (0.06) | -0.014 (-0.032 - 0.004) | 0.538 |
| Splenium of corpus callosum left | | 0.56 (0.05) | 0.51 (0.07) | 0.54 (0.08) | 0.54 (0.07) | -0.012 (-0.038 - 0.015) | 0.653 |
| Superior cerebellar peduncle left | | 0.50 (0.04) | 0.51 (0.03) | 0.48 (0.09) | 0.48 (0.08) | -0.016 (-0.041 - 0.01) | 0.582 |
| Superior cerebellar peduncle right | | 0.51 (0.04) | 0.50 (0.03) | 0.49 (0.07) | 0.49 (0.09) | -0.014 (-0.038 - 0.01) | 0.582 |
| Superior corona radiata left | | 0.41 (0.03) | 0.42 (0.03) | 0.42 (0.04) | 0.41 (0.05) | -0.007 (-0.021 - 0.008) | 0.653 |
| Superior corona radiata right | | 0.44 (0.03) | 0.45 (0.04) | 0.44 (0.04) | 0.43 (0.06) | -0.013 (-0.029 - 0.002) | 0.46 |
| Superior fronto-occipital fasciculus (could be a part of anterior internal capsule) left | | 0.47 (0.04) | 0.46 (0.05) | 0.46 (0.05) | 0.44 (0.08) | -0.022 (-0.044 - 0) | 0.384 |
| Superior fronto-occipital fasciculus (could be a part of anterior internal capsule) right | | 0.47 (0.05) | 0.45 (0.07) | 0.46 (0.06) | 0.45 (0.09) | -0.013 (-0.039 - 0.013) | 0.604 |
| Superior longitudinal fasciculus left | | 0.37 (0.04) | 0.36 (0.04) | 0.36 (0.05) | 0.37 (0.04) | -0.008 (-0.023 - 0.008) | 0.604 |
| Superior longitudinal fasciculus right | | 0.39 (0.04) | 0.38 (0.04) | 0.39 (0.05) | 0.38 (0.05) | -0.012 (-0.028 - 0.005) | 0.582 |
| Tapatum left | | 0.33 (0.09) | 0.30 (0.07) | 0.32 (0.07) | 0.33 (0.10) | -0.01 (-0.042 - 0.022) | 0.843 |
| Tapatum right | | 0.33 (0.08) | 0.26 (0.07) | 0.30 (0.08) | 0.32 (0.09) | 0 (-0.03 - 0.03) | 0.996 |
| Uncinate fasciculus left | | 0.32 (0.06) | 0.33 (0.07) | 0.33 (0.07) | 0.33 (0.05) | 0.005 (-0.02 - 0.031) | 0.892 |
| Uncinate fasciculus right | | 0.31 (0.04) | 0.29 (0.05) | 0.31 (0.05) | 0.30 (0.07) | 0.005 (-0.016 - 0.026) | 0.892 |
| MD (mm^2^/s) (*10^-3^) | | Mean (SD) | Mean (SD) | Mean (SD) | Mean (SD) |  |  |
| Anterior corona radiata left | | 0.77 (0.05) | 0.79 (0.06) | 0.77 (0.03) | 0.78 (0.06) | 7.24 (-8.83 - 23.31) | 0.653 |
| Anterior corona radiata right | | 0.79 (0.05) | 0.83 (0.07) | 0.79 (0.05) | 0.80 (0.05) | -0.33 (-19.06 - 18.4) | 0.993 |
| Anterior limb of internal capsule left | | 0.74 (0.05) | 0.75 (0.06) | 0.73 (0.07) | 0.76 (0.06) | 12.2 (-8.84 - 33.25) | 0.582 |
| Anterior limb of internal capsule right | | 0.72 (0.06) | 0.74 (0.08) | 0.71 (0.06) | 0.73 (0.05) | 3.46 (-20.29 - 27.22) | 0.898 |
| Body of corpus callosum left | | 1.13 (0.12) | 1.19 (0.15) | 1.15 (0.15) | 1.14 (0.13) | 5.63 (-42.61 - 53.86) | 0.915 |
| Cerebral peduncle left | | 1.02 (0.13) | 1.01 (0.10) | 1.04 (0.15) | 1.00 (0.11) | 8.73 (-40.64 - 58.1) | 0.892 |
| Cerebral peduncle right | | 0.97 (0.13) | 0.97 (0.14) | 0.98 (0.12) | 0.95 (0.10) | 7.46 (-38.13 - 53.05) | 0.892 |
| Cingulum (Caudal anterior) left | | 0.76 (0.05) | 0.79 (0.08) | 0.77 (0.09) | 0.77 (0.08) | 12.93 (-17.73 - 43.58) | 0.684 |
| Cingulum (Caudal anterior) right | | 0.76 (0.05) | 0.79 (0.08) | 0.76 (0.07) | 0.77 (0.06) | 0.92 (-22.9 - 24.75) | 0.993 |
| Cingulum (hippocampus) left | | 0.90 (0.09) | 0.88 (0.09) | 0.91 (0.14) | 0.88 (0.07) | 0.82 (-38.98 - 40.62) | 0.993 |
| Cingulum (hippocampus) right | | 0.91 (0.10) | 0.88 (0.09) | 0.93 (0.14) | 0.87 (0.10) | -8.28 (-52.05 - 35.5) | 0.892 |
| Corticospinal tract left | | 1.17 (0.20) | 1.15 (0.18) | 1.20 (0.28) | 1.14 (0.17) | -14.79 (-101.6 - 72.02) | 0.892 |
| Corticospinal tract right | | 1.20 (0.20) | 1.15 (0.20) | 1.21 (0.32) | 1.15 (0.17) | -14.35 (-106.86 - 78.16) | 0.892 |
| External capsule left | | 0.80 (0.05) | 0.79 (0.03) | 0.79 (0.05) | 0.80 (0.04) | 14.52 (-2.11 - 31.16) | 0.46 |
| External capsule right | | 0.77 (0.05) | 0.76 (0.04) | 0.76 (0.05) | 0.78 (0.05) | 12.5 (-6.35 - 31.35) | 0.582 |
| Fornix (column and body of fornix) left | | 1.95 (0.38) | 2.03 (0.37) | 1.94 (0.44) | 1.83 (0.35) | -100.85 (-222.52 - 20.82) | 0.502 |
| Fornix (cres) / Stria terminalis (can not be resolved with current resolution) left | | 1.22 (0.21) | 1.31 (0.19) | 1.27 (0.36) | 1.20 (0.15) | -17.38 (-97.12 - 62.37) | 0.892 |
| Fornix (cres) / Stria terminalis (can not be resolved with current resolution) right | | 1.11 (0.19) | 1.15 (0.17) | 1.15 (0.35) | 1.08 (0.17) | -27.91 (-111.47 - 55.65) | 0.809 |
| Genu of corpus callosum left | | 0.97 (0.10) | 1.02 (0.10) | 0.97 (0.11) | 0.95 (0.08) | -18.97 (-53.03 - 15.09) | 0.582 |
| Inferior cerebellar peduncle left | | 0.90 (0.12) | 0.88 (0.08) | 0.94 (0.18) | 0.89 (0.12) | 11.35 (-40.93 - 63.63) | 0.892 |
| Inferior cerebellar peduncle right | | 0.94 (0.13) | 0.94 (0.10) | 1.00 (0.14) | 0.94 (0.11) | 25.09 (-21.83 - 72.01) | 0.582 |
| Medial lemniscus left | | 0.80 (0.08) | 0.77 (0.06) | 0.87 (0.39) | 0.79 (0.06) | 22.48 (-62.92 - 107.89) | 0.87 |
| Medial lemniscus right | | 0.78 (0.07) | 0.78 (0.06) | 0.83 (0.22) | 0.82 (0.17) | 59.59 (0.79 - 118.4) | 0.384 |
| Middle cerebellar peduncle | | 1.07 (0.10) | 1.03 (0.09) | 1.07 (0.18) | 1.05 (0.12) | -1.75 (-52.84 - 49.33) | 0.993 |
| Pontine crossing tract (a part of MCP) | | 0.68 (0.05) | 0.69 (0.08) | 0.74 (0.31) | 0.72 (0.10) | 43.82 (-23.87 - 111.52) | 0.582 |
| Posterior corona radiata left | | 0.79 (0.06) | 0.81 (0.06) | 0.80 (0.06) | 0.80 (0.06) | 16.79 (-3.71 - 37.29) | 0.502 |
| Posterior corona radiata right | | 0.77 (0.05) | 0.79 (0.05) | 0.78 (0.05) | 0.79 (0.05) | 23.68 (6.64 - 40.71) | 0.32 |
| Posterior limb of internal capsule left | | 0.70 (0.03) | 0.69 (0.03) | 0.70 (0.05) | 0.71 (0.04) | 16.32 (0.46 - 32.18) | 0.384 |
| Posterior limb of internal capsule right | | 0.65 (0.03) | 0.64 (0.03) | 0.65 (0.04) | 0.66 (0.03) | 12.88 (-0.29 - 26.05) | 0.398 |
| Posterior thalamic radiation (include optic radiation) left | | 0.82 (0.15) | 0.84 (0.08) | 0.81 (0.09) | 0.82 (0.08) | 4.31 (-34.64 - 43.26) | 0.915 |
| Posterior thalamic radiation (include optic radiation) right | | 0.83 (0.08) | 0.87 (0.07) | 0.83 (0.06) | 0.86 (0.10) | 6.5 (-21.44 - 34.43) | 0.892 |
| Retrolenticular part of internal capsule left | | 0.80 (0.09) | 0.83 (0.10) | 0.81 (0.08) | 0.79 (0.06) | 4.56 (-23.96 - 33.08) | 0.892 |
| Retrolenticular part of internal capsule right | | 0.72 (0.06) | 0.74 (0.05) | 0.73 (0.05) | 0.73 (0.04) | 10.47 (-8.83 - 29.77) | 0.582 |
| Sagittal stratum (include inferior longitidinal fasciculus and inferior fronto-occipital fasciculus) left | | 0.82 (0.08) | 0.83 (0.06) | 0.84 (0.10) | 0.82 (0.05) | 16.45 (-11.04 - 43.94) | 0.582 |
| Sagittal stratum (include inferior longitidinal fasciculus and inferior fronto-occipital fasciculus) right | | 0.80 (0.08) | 0.82 (0.07) | 0.82 (0.10) | 0.82 (0.07) | 16.16 (-11.79 - 44.1) | 0.582 |
| Splenium of corpus callosum left | | 0.91 (0.10) | 0.97 (0.09) | 0.93 (0.12) | 0.93 (0.09) | 10.56 (-24.11 - 45.23) | 0.843 |
| Superior cerebellar peduncle left | | 1.08 (0.11) | 1.08 (0.15) | 1.11 (0.14) | 1.10 (0.16) | 7.29 (-46.8 - 61.38) | 0.905 |
| Superior cerebellar peduncle right | | 1.08 (0.11) | 1.12 (0.15) | 1.08 (0.10) | 1.10 (0.15) | -5.87 (-53.83 - 42.09) | 0.915 |
| Superior corona radiata left | | 0.74 (0.04) | 0.75 (0.04) | 0.75 (0.07) | 0.77 (0.08) | 26.1 (3.71 - 48.48) | 0.384 |
| Superior corona radiata right | | 0.71 (0.04) | 0.73 (0.06) | 0.71 (0.07) | 0.74 (0.08) | 22.57 (-2.2 - 47.35) | 0.46 |
| Superior fronto-occipital fasciculus (could be a part of anterior internal capsule) left | | 0.74 (0.06) | 0.78 (0.11) | 0.74 (0.07) | 0.79 (0.15) | 29.49 (-3.42 - 62.4) | 0.46 |
| Superior fronto-occipital fasciculus (could be a part of anterior internal capsule) right | | 0.73 (0.14) | 0.81 (0.19) | 0.74 (0.12) | 0.78 (0.17) | 15.6 (-41.42 - 72.62) | 0.863 |
| Superior longitudinal fasciculus left | | 0.77 (0.06) | 0.78 (0.06) | 0.78 (0.07) | 0.78 (0.05) | 13.62 (-7.44 - 34.67) | 0.582 |
| Superior longitudinal fasciculus right | | 0.74 (0.06) | 0.75 (0.06) | 0.74 (0.06) | 0.76 (0.06) | 20.05 (-2.97 - 43.06) | 0.46 |
| Tapatum left | | 1.70 (0.36) | 1.82 (0.28) | 1.65 (0.36) | 1.72 (0.45) | 4.2 (-137.63 - 146.03) | 0.993 |
| Tapatum right | | 1.73 (0.33) | 2.02 (0.25) | 1.79 (0.39) | 1.74 (0.43) | -25.18 (-157.53 - 107.16) | 0.892 |
| Uncinate fasciculus left | | 0.86 (0.14) | 0.85 (0.09) | 0.84 (0.12) | 0.84 (0.06) | -12.49 (-56.68 - 31.7) | 0.863 |
| Uncinate fasciculus right | | 0.83 (0.09) | 0.87 (0.15) | 0.80 (0.07) | 0.85 (0.14) | -4.07 (-48.56 - 40.42) | 0.936 |
